# Quality of life during 5 years after stereotactic radiotherapy in stage I non-small cell lung cancer

**DOI:** 10.1186/s13014-015-0405-9

**Published:** 2015-04-22

**Authors:** Rutger J Ubels, Sahar Mokhles, Eleni R Andrinopoulou, Cornelia Braat, Noëlle C van der Voort van Zyp, Shafak Aluwini, Joachim G J V Aerts, Joost J Nuyttens

**Affiliations:** Department of Radiation Oncology, Erasmus MC Cancer Institute, Postbus 2040, 3000 Rotterdam, CA the Netherlands; Department of Pulmonology, Erasmus MC Cancer Institute, Rotterdam, the Netherlands; Department of Cardio-Thoracic Surgery, Erasmus MC, Rotterdam, the Netherlands; Department of Biostatistics, Erasmus MC, Rotterdam, the Netherlands

## Abstract

**Purpose:**

To determine the long-term impact of stereotactic radiotherapy (SRT) on the quality of life (QoL) of inoperable patients with early-stage non-small cell lung cancer (NSCLC).

**Methods and materials:**

From January 2006 to February 2008, 39 patients with pathologically confirmed T1-2N0M0 NSCLC were treated with SRT. QoL, overall survival and local tumor control were assessed. The European Organization for Research and Treatment of Cancer Quality of Life Questionnaire (EORTC QLQ)-C30 and the lung cancer-specific questionnaire QLQ-LC13 were used to investigate changes in QoL. Assessments were done before treatment, at 3 weeks, every 2–3 months during the first two years, and then every 6 months until 5 years after the treatment or death or progressive disease. The median follow up was 38 months.

**Results:**

During the 5 years after treatment with SRT for stage I NSCLC, the level of QoL was maintained: There was a slow decline (slope: −0.015) of the global health status over the 5 years (p < 0.0001). The physical functioning and the role functioning improved slowly (slope: 0.006 and 0.004, resp.) over the years and this was also significant (p < 0.0001). The emotional functioning (EF) improved significantly at 1 year compared to the baseline. Two years after the treatment dyspnea slowly increased (slope: 0.005, p = 0.006). The actuarial overall survival was 62% at 2 years and 31% at 5-years.

**Conclusion:**

QoL was maintained 5 years after SRT for stage I NSCLC and EF improved significantly. Dyspnea slowly increased 2 years after the treatment.

## Background

Stereotactic radiotherapy (SRT) has proved to be a good alternative treatment to surgery for medically inoperable patients with early stage non-small cell lung cancer (NSCLC). Prospective trials evaluating the use of SRT showed excellent local tumor control rates (78%–97%) [1]. Overall survival, while more variable, has improved compared to historical controls [1,2]. The treatment is well tolerated, even in elderly patients [3,4]. An essential goal in any cancer treatment is to maintain or improve the patients’ quality of life (QoL). However, only a few publications have evaluated the impact of treatment on the patients’ QoL. SRT does not lead to significant worsening of health related quality of life (HRQoL) in the first year after treatment. Patients referred for SRT have substantially worse baseline HRQoL scores than those reported in the surgical literature and clinically relevant deteriorations in HRQoL subscale scores were not observed after SRT [5]. QoL was evaluated in medically inoperable patients with NSCLC treated either with SRT or conventional three-dimensional conformal radiotherapy. At one year patients treated with SRT had a stable global QoL and physical functioning (PF)and dyspnea, while patients treated with 3D-CRT had a decreased PF approaching clinical significance [6]. In 2009 we published the results of the QoL one year after treatment with SRT, using the European Organization for Research and Treatment of Cancer (EORTC) quality of life questionnaire (QLQ) C30 and lung cancer-specific supplementary questionnaire QLQ LC13. QoL was maintained and the emotional functioning (EF) improved significantly. Other function scores and QLQ-C30 and QLQ-LC13 lung symptoms (such as dyspnea and coughing) showed no significant changes [7]. To our knowledge, this is the first study to report the outcome of QoL 5 years after SRT for patients with stage I NSCLC.

## Methods

### Patients and treatment

Between January 2006 and February 2008, 43 patients who refused surgery or had an inoperable stage T1-2N0M0 NSCLC entered our prospective phase II trial. The trial was accepted by the Medical Ethical Committee of the Erasmus Medical Center (METC Erasmus-MC number: 2005–300) and was in agreement with the Declaration of Helsinki. Pathological confirmation of malignancy was obtained for all patients. Diagnostic staging included computed tomography (CT) scanning of all patients and positron emission tomography (PET) scanning for all but 4 patients. Four patients were excluded from analysis due to a lack of pretreatment assessment (n = 2), progressive disease 3 weeks after treatment (n = 1), and 1 patient declined to participate after inclusion. Comorbidity was registered using the Charlson comorbidity index and the cumulative illness ranking score [8,9]. Patient characteristics are listed in Table 1. One patient included in this analysis had a T2 tumor at the time of inclusion but a T3 tumor at the time of treatment.

**Table 1:** Patient and tumor characteristics*

**Characteristic**	**No. of patients (% of total)**
Medically inoperable	33 (85)
Refused surgery	6 (15)
Charlson Comorbidity Score	
0-2	20 (51)
03-apr	13 (33)
<3	6 (15)
Median Cumulative Illness Ranking (range)	6 (2–16)
Incidence of COPD	22 (56)
Tumor location	
Peripheral	33 (85)
Central	6 (15)
T-classification	
T1	17 (44)
T2	21 (54)
T3	1 (3)
Histology	
Squamous cell carcinoma	14 (36)
Large cell carcinoma	13 (33)
Adenocarcinoma	8 (21)
Other	4 (10)
PTV median (cc) (range)	46 (7–609)

*Median age, 77 years (range, 55–87 years). No: number; PTV: planning Target Volume.

All patients were treated with real-time tumor tracking using the CyberKnife [10]. The technique has been described previously [11]. Treatment consisted of 60 Gy in 3 fractions for 30 patients. A risk-adaptive treatment schedule consisting of 48 to 50 Gy in 5 to 6 fractions was used to treat 6 patients with central tumors and 1 patient with a large T2 tumor. Two patients were treated with 45 Gy in 3 fractions by choice of the treating physician. Treatment dose was prescribed to the 78 to 87% isodose line, covering at least 95% of the planning target volume (PTV). The maximum dose was defined by the 100% isodose line. Treatment planning was done with the On Target treatment planning system version 3.4.1 (Accuray Inc., Sunnyvale, CA). Correction for tissue inhomogeneity was achieved by using the equivalent path length algorithm. None of the patients were treated with chemotherapy prior to treatment or in an adjuvant setting.

### QoL instruments

QoL assessments were performed before treatment, at 3 weeks, and at 2, 4, 6, 9, 12, 15, 18, 21 and 24 months after the treatment. After 24 months, the assessments were performed every 6 months until 5 years after the treatment or death or progressive disease. Patients with evidence of progressive disease were excluded from further analysis to prevent bias caused by disease progression or treatment of progressive disease. QoL was evaluated by means of the European Organization for Research and Treatment of Cancer (EORTC) core questionnaire, Quality of Life Questionnaire (QLQ) C30 (version 3.0), and supplementary lung cancer-specific module QLQ-LC13. The QLQ-C30 is a 30-item questionnaire composed of five functional scales, three symptom scales, a global health status/QoL scale, and six single items. The single items assess additional symptoms commonly reported by cancer patients. This questionnaire has proven to be a valid and reliable tool when used among a wide range of cancer patient populations, including lung cancer patients [12]. The lung cancer module is designed for patients with various disease stages treated with chemotherapy and/or radiotherapy. It consists of 13 questions assessing lung cancer-associated symptoms, treatment-related side effects, and pain medication. The EORTC QoL and symptom measures were rescaled to percentages (scores 0 to 100%) through linear transformation. A high score for the function and QoL scales represents a high level of functioning/high QoL, whereas a high symptom score represents a high level of symptoms. The questionnaires have been translated and validated for use in a Dutch population.

### Follow-up and toxicity scoring

The first clinical examination was performed 3 weeks after SRT. Clinical follow-up was performed every 3 months, and a CT scan was performed at 2, 4 and 6 months, and every 3 months thereafter. After 2 years it was performed every half year up to 5 years. The patient’s physician scored the toxicity at each-out patient visit, using common terminology criteria for adverse events version 3.0. There was acute toxicity if it occurred within 4 months and late toxicity if it occurred thereafter.

### Statistical analyses

The data in the present study were analyzed with mixed-effects models to evaluate changes over time in the mean QoL and symptom scores. Mixed-effects models are an appropriate tool for the analysis of dependent data such as data collected in a hierarchical manner, e.g. when a number of observations are collected over time on the same patient [13,14]. The advantage of using mixed-effects models is that they model the evolution of a longitudinal outcome over time while accounting for the correlation between repeated measurements in each patient. Moreover, these models are able to deal with unbalanced data, that is when the number of observations per individual is not the same, or when time between repeated measurements of each individual varies. Specifically, mixed-effects models consist of the fixed and the random effects. The fixed effects describe the average evolution in time of a specific longitudinal outcome (e.g. one of the five functional scales), while the random effects describe the evolution in time of each patient. Due to heterogeneity in the residuals plot, the logarithmic scale was used for some variables. Missing values due to non-response of questionnaire were assumed to be missing at random, which means that the missing value was assumed to be independent of the unobserved measurement [14,15]. All analyses were performed with the R statistical software (version 2.13.2, 2011. R Development Core Team 2011, R Foundation for Statistical Computing, Vienna, Austria). All statistical tests with a P-value of 0.05 or lower were considered significant. Overall survival was measured from the start of radiotherapy until death by any cause. Patients still alive at the date of last contact were censored. Local tumor control was calculated from the first day of treatment until the diagnosis of a local recurrence. Patients without a local recurrence were censored on the last day of contact. In the absence of biopsy confirmed viable carcinoma, local recurrence was defined as a 20% increased longest-tumor dimension on the CT scan compared to the previous CT scan. In addition, a corresponding avid lesion on the PET scan was required.

## Results

### Compliance with QoL assessments

QoL was assessed in 39 patients. The mean compliance over the 5 years was more than 93% (range, 78-100%). At 5 years 10 patients were still alive without progression. The details are shown in Table 2.

**Table 2:** **Compliance with quality of life assessments**

**Time (months)**	**Compliance (%)**	**Nr. of patients still alive without progression**
0.75 (3 weeks)	90	35/39
2	95	35/37
4	95	35/37
6	100	36/36
9	96	27/28
12	95	20/21
15	95	19/20
18	100	20/20
21	95	19/20
24	95	19/20
30	95	18/19
36	78	14/18
42	87	13/15
48	86	12/14
60	100	10/10

### QoL and baseline symptoms

**Figure 1:**
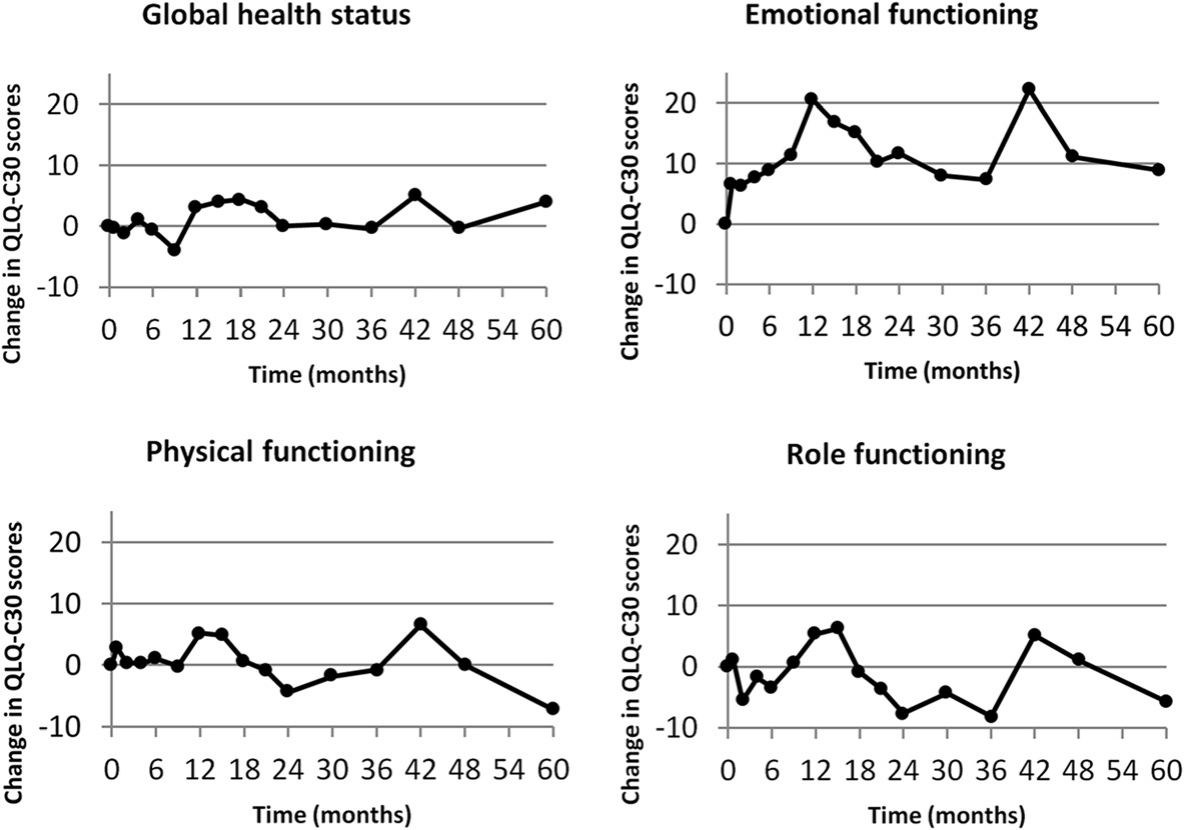
Change in mean global health and functional scores.

**Figure 2:**
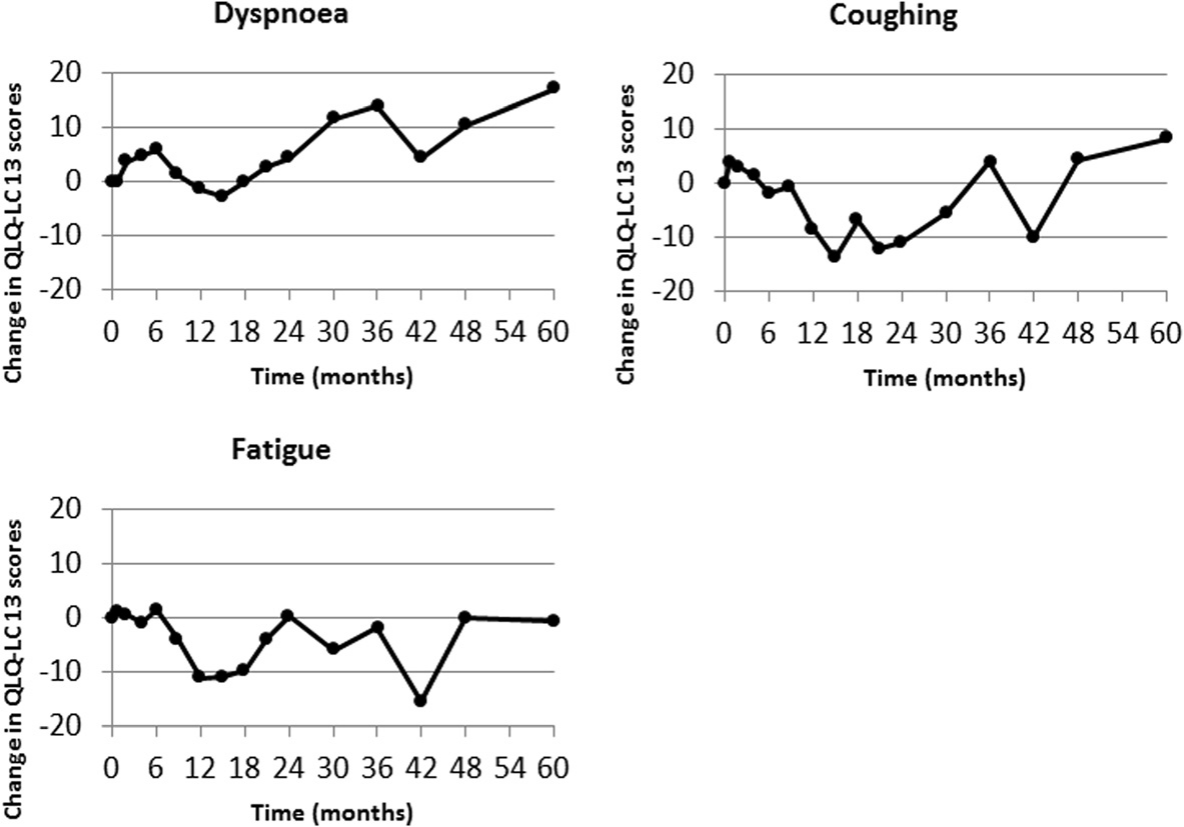
Change in mean QLQ-LC13 dyspnea, coughing and fatigue scores.

Changes in QLQ-C30 mean global health status (GH) and function scores (EF, PF and RF) during follow-up are shown in Figure 1. Changes in QLQ-LC13 mean symptom scores (dyspnea, coughing and fatigue) are shown in Figure 2. During the first year, the global health status was near the baseline value, improved to a score of 4 at 18 months and then significantly declined (slope: −0.015) to the baseline value during the next years. The PF score as well as the role functioning (RF) significantly improved slowly (slope: 0.006 and 0.004, resp.) over the years. Due to the fluctuation of the EF score over the 5 years, the changes over time were not significant, but the mean EF score at 1 year was significantly different compared to the pretreatment score (p = 0.0003). The small rise (slope: 0.004) over time in the cognitive functioning was also significant (p = 0.004), but not the social functioning.

The dyspnea score increased during the first 6 months to a score of 6, then ameliorated after the 1st year. During the following years, the dyspnea score gradually increased to a score of 17 at 5 years. This increase (slope: 0.005) over time was significant (p = 0.006) for the data from the QLQ-C30 but not for the data from the QLQ-LC13. The coughing score increased to 4 at 3 weeks after the treatment and slowly decreased during the first 2 years to −11. After the first 2 years, the score increased to a score of 8 at 5 years (p = 0.57). The fatigue score at one year decreased to a score of −10, but raised thereafter to a score of −0.6 at 5 years. This slow increase (slope: 0.003) over the 5 years (p = 0.05) was significant.

### Overall survival and local tumor control

The overall survival rate was 62% at 2 year and 31% at 5 years. Twenty-seven patients died; 12 patients died from metastatic disease, and 15 patients died from intercurrent disease. Causes of intercurrent death are shown in Table 3. Local tumor control was 97% at 2 years and 93% at 5 years. Two patients had a local recurrence. The disease free survival was 69% at 2 years and 52% at 5 years.

**Table 3:** **Causes of death**

**Cause of death**	**Nr. of patients (Total 27)**
Metastatic disease	12
Intercurrent	15
cardiovascular	3
Mortality during surgery	1
Sudden death of unknown cause	3
General deterioration	6
Pulmonal infection	2

Fourteen patients had distant metastases. Of the 14 patients with distant metastases, 6 patients had mediastinal lymph nodes. There were no patients with isolated regional recurrence. The median follow-up was 38 months (range, 4–71 months).

### Toxicity

Treatment related grade 4 or 5 toxicity didn’t occur. Twelve patients had no acute side effects at all. The most common grade 1 and 2 toxicities were respiratory (dyspnea and coughing). Acute grade 2 toxicity involved 12 patients, of which 6 with dyspnea, 1 with esophageal pain, 1 with thoracic pain and 4 with coughing. There were 14 patients with late grade 2 toxicity: dyspnea and thoracic pain occurred both in 6 patients and chronic cough in 2. Two patients had acute grade 3 toxicity, 1 with dyspnea and 1 with thoracic pain. Late grade 3 toxicity occurred in 4 patients, 2 with dyspnea and 2 with thoracic pain.

## Discussion

We observed that QoL was maintained 5 years after SRT. The global health increased during the first 1.5 years but decreased thereafter to the baseline. The PF and RF significantly improved slowly, although the improvements were small. The EF improved significantly in the first year, but declined thereafter. Respiratory symptoms (dyspnea and coughing) did not get worse in the first two years, although it slowly increased in the next years after SRT.

Four other studies have reported health related quality of life outcomes (HRQoL) after SRT in patients with early-stage NSCLC [2,5,6,16]. These studies report on the QoL one to three years after the SBRT. Widder et al. investigated changes of HRQoL parameters after SRT (202 patients) and 3-D treatment (27 patients) in two prospective cohorts of inoperable patients. In all studies, global QoL and PF were stable after treatment, no statistically or clinically significant worsening of any of the HRQoL functioning or symptom scores at any follow-up time point was observed in our and other mentioned studies [5,6,16]. Most noticeable difference is that our study showed a statistically significant improvement of the EF at 1 year. Mathieu et al. did report a trend in QLQ-C30 emotional score improvement of 14 at 36 months [16].

A prospective study with patients diagnosed with early stage lung cancer undergoing 3D-CRT showed a gradual and significant increase in dyspnea, fatigue, and appetite loss, together with a significant deterioration of RF compared to the base line measurement. The global QoL did not deteriorate, EF did not improve. Their hypothesis for worsening of dyspnea and fatigue was because of pre-existing, slowly progressive chronic obstructive pulmonary disease (COPD) and radiation-induced pulmonary changes [17].

Langendijk et al. investigated the effect of respiratory symptoms on QoL in patients with stage I-III lung cancer during the first 2 years after the treatment. At the base line, dyspnea was the most important and significant respiratory symptom affecting all EORTC scales, with the exception of EF [18].

In comparison with surgery, the HRQoL after stereotactic radiotherapy compared to HRQoL after the surgery is at 3 or 6 months after the treatment in general better. Poghosyan et al. reviewed 19 out of 337 studies and concluded that participants had worse physical function at 6-months after surgery and had decreased physical function up to 2-years after surgery, compared to the pre-surgical status. Pain, fatigue, dyspnea and coughing were the most prevalent symptoms. Increased levels of dyspnea and fatigue persisted for at least 2-years after surgery. Kenny at al. who studied the HRQoL in 173 patients with stage I and II NSCLC reported that surgery substantially reduced HRQoL across all dimensions except emotional functioning. HRQoL improved in the 2 years after surgery for patients without disease recurrence, although approximately half continued to experience symptoms and functional limitations.

There is not much known about the quality of life more than 2 years after the treatment in patients with early stage lung cancer. It is generally known that patients with COPD have a decline of their long function over time. This is mainly based on the study of Fletcher and Peto [19]. More than 35 years ago, they did report on the natural history of tobacco smoke–related chronic airflow obstruction. Fletcher and Peto measured the forced expiratory volume in 1 second (FEV1) every 6 months for an 8-year follow-up period in a cohort of 792 working men and concluded that a lower FEV1 declined greater for similar intervals of time in COPD patients who smoked. However recent research found that patients with COPD GOLD stage I had a decline of about 40 ml/year, patients with COPD GOLD stage II a decline of 47–79 ml/year, patients with COPD GOLD stage III, a decline of 56–59 ml/year, and patients with COPD GOLD stage IV a decline of <35 ml/year [20,21]. Many of our patients had COPD. The dyspnea score increased during the first 6 months to a score of 6, then ameliorated after the 1st year (score −3). During the following years, the dyspnea score gradually increased to a score of 17 at 5 years. So the increase of the dyspnea score after the 1st year can be related to decline of the lung function over time or due to the radiotherapy. Probably it is caused by both. Several studies did report on the QoL in patients with COPD. Carrasco Garrido et al. did report on the HRQoL in 10711 patients and concluded that patients with stable COPD stages 2–4 did show a reduction of their HRQoL, even in mild stages of the disease. The factors determining the HRQoL include sex, FEV1, use of oxygen therapy, and number of visits to emergency rooms and hospital admissions [22]. Bridevaux et al. studied 519 patients with COPD GOLD stage I and concluded also that these patients have a lower QoL than the 3627 asymptomatic subjects with normal lung function. The slow decline of the global health (GH) score over the last 3 years is maybe caused by the decrease of the lung function and increase of dyspnea in our patients, but the PF score as well as the RF ameliorated slowly over the years. However, the impact in COPD in patients with lung cancer is not completely clear [23]. Mohan et al. studied 160 patients with COPD and stage III and IV lung cancer and concluded that no significant differences were found in clinical profile, Karnofsky performance status, or QoL scores between patients with and without COPD [24]. On the other hand, Gore et al. compared the QoL in end-stage COPD patient with NSCLC patients and concluded that the end-stage COPD patients experienced a poor HRQoL comparable to or worse than that of advanced NSCLC patients [25].

The actuarial overall survival of our study was 62% at 2 years and 31% at 5 years. The actuarial local tumor control was 97% at 2 years and 93% at 5 years. This is in agreement with other studies: Widder et al. reported estimates at two years for 3D-CRT versus SRT of 48% versus 72% for overall survival (OS),and 78% versus 95% for local control (LC), respectively [6]. In the patient-report of Lagerwaard et al. HRQoL data were collected prospectively in 382 consecutive patients treated with SRT. The median survival was 40 months, with a 2-year OS of 66% [5].

In our study the overall compliance was more than 93%, so the missing data of QoL assessments is minimal. Though, the major limitation of this study was the small number of patients with increasing follow-up time. Therefore the study has not enough power and should be seen as descriptive, as this is the first report about QoL during 5-years. More research will be needed, especially a bigger number of patients for more data.

## Conclusions

During the 5 years after treatment with stereotactic radiotherapy for stage I NSCLC, the level of QoL was maintained: There was a slow decline of the Global Health status over the 5 years (p < 0.0001). The physical functioning score as well as the role function score did ameliorated slowly over the years and this was also significant (p < 0.0001). The emotional functioning improved significantly at 1 year compared to the base line. Two years after the treatment, the dyspnea slowly increased.

## Abbreviations

SRT: Stereotactic radiotherapy
QoL: Quality of life
NSCLC: Non-small cell lung cancer
EORTC: European organization for research and treatment of cancer
QLQ: Life questionnaire
EF: Emotional functioning
HRQoL: Health related quality of life
PH: Physical functioning
METC: Medical ethical committee
CT: Computed tomography
PET: Positron emission tomography
PTV: Planning target volume
RF: Role functioning
FEV1: Forced expiratory volume in 1 second
GH: Global health
OS: Overall survival
LC: Local control
